# Increase in circulating Foxp3+CD4+CD25^high^ regulatory T cells in nasopharyngeal carcinoma patients

**DOI:** 10.1038/sj.bjc.6603580

**Published:** 2007-01-30

**Authors:** K-M Lau, S H Cheng, K W Lo, S A K W Lee, J K S Woo, C A van Hasselt, S P Lee, A B Rickinson, M H L Ng

**Affiliations:** 1 Department of Anatomical and Cellular Pathology, The Chinese University of Hong Kong, Hong Kong, China; 2 State Key Laboratory in Oncology in South China, The Chinese University of Hong Kong, Hong Kong, China; 3 Li Ka Shing Institute of Health Science, The Chinese University of Hong Kong, Hong Kong, China; 4 Division of Otorhinolaryngology, Department of Surgery, Prince of Wales Hospital, The Chinese University of Hong Kong, Hong Kong, China; 5 Cancer Research UK, Institute for Cancer Studies, University of Birmingham, Birmingham, UK

**Keywords:** nasopharyngeal carcinoma, Treg, antitumour immunity, tumour infiltrating lymphocytes

## Abstract

Nasopharyngeal carcinoma (NPC) is an Epstein–Barr virus-associated disease with high prevalence in Southern Chinese. Using multiparametric flow cytometry, we identified significant expansions of circulating naïve and memory CD4+CD25^high^ T cells in 56 NPC patients compared with healthy age- and sex-matched controls. These were regulatory T cells (Treg), as they overexpressed Foxp3 and GITR, and demonstrated enhanced suppressive activities against autologous CD4+CD25− T-cell proliferation in functional studies on five patients. Abundant intraepithelial infiltrations of Treg with very high levels of Foxp3 expression and absence of CCR7 expression were also detected in five primary tumours. Our current study is the first to demonstrate an expansion of functional Treg in the circulation of NPC patients and the presence of infiltrating Treg in the tumour microenvironment. As Treg may play an important role in suppressing antitumour immunity, our findings provide critical insights for clinical management of NPC.

Nasopharyngeal carcinoma (NPC) is an Epstein–Barr virus (EBV)-associated malignant disease with high prevalence in Southern Chinese. Despite favourable responses to radio-chemotherapy in most patients with early disease, a significant number of patients present with metastatic or refractory disease or develop relapses (Teo *et al*, 2006). Thus, alternative therapeutic approaches need to be established. One approach is the development of immunotherapy specifically targeting NPC cells for immune destruction by EBV-specific cytotoxic T lymphocytes. Thus far, limited studies reported variable clinical responses (Chua *et al*, 2001; Comoli *et al*, 2005; Straathof *et al*, 2005).

CD4+CD25+ regulatory T cells (Treg) can suppress the activation and proliferation of CD4+ and CD8+ T cells (Piccirillo and Shevach, 2001; Woo *et al*, 2002) and play an important role in controlling autoreactive T cells. However, for several types of human cancer, an increase of Treg in the peripheral blood (PB) and the presence of Treg at the tumour site has been reported (Woo *et al*, 2001, 2002; Liyanage *et al*, 2002; Wolf *et al*, 2003; Ormandy *et al*, 2005; Schaefer *et al*, 2005), indicating that these cells may also suppress T-cell-mediated antitumour immunity (Piccirillo and Shevach, 2001; Curiel *et al*, 2004). Understanding the role of Treg in NPC patients has important implications for disease management, including the development of successful T-cell immunotherapy.

## MATERIALS AND METHODS

### Patient recruitment

With informed consent, a total of 57 poorly differentiated and undifferentiated NPC patients were prospectively recruited at diagnosis at the Prince of Wales Hospital (2005–2006), including four patients with stage I tumour, 18 with stage II, 17 with stage III, and 18 with stage IV tumours. All of the tumours were confirmed to be EBV-positive by EBER *in situ* hybridisation. Five patients were recruited for tumour biopsies collection. For control group, 56 sex- and age-matched healthy blood donors from the Hong Kong Red Cross Blood transfusion Service were used.

### Multicolour flow cytometric analysis

Peripheral blood mononuclear cells (PBMNC) were isolated from freshly drawn blood by centrifugation on Ficoll–Hypaque gradient solution (Pharmacia, Uppsala, Sweden). Tumour biopsies were dissociated into single cell suspensions with collagenase digestion and/or mechanical dissociation. The PBMNC and suspended cells were immunostained with fluorochrome-conjugated anti-human antibodies for 25 min at 4°C. For intracellular antigens, the cells were fixed and permeabilised with Becton Dickinson™ (BD) fix/perm solution (BD Biosciences, Bedford, MA, USA) before immunostaining. The panel of antibody combinations conjugated to fluorescein isothiocyanate (FITC), phycoerythrin (PE), phycoerythrin-cyanin 5 (PECy5), and allophycocyanin (APC)/AlexaFluor647 (AF647) were (1) CD25-FITC/CD3-PE/CD4-PECy5/CD8-APC, (2) CD25-FITC/CD45RO-PE/CD4-PECy5, (3) CD25-FITC/CCR7-PE/CD4-PECY5/CD152-APC, and (4) CD25-FITC/GITR-PE/CD4-PECy5/Foxp3-AF647. Cell acquisition was performed using a BD FACSCalibur flow cytometer. Data of ⩾200 000 lymphocytes gated by forward and side scatter properties were collected for the examination of various marker expressions in the cells. Flow data were analysed using FlowJo^©^ Analysis Software (Treestar Inc., Ashland, OR, USA) for gating different populations of T-cell subsets and determination of expression levels of various markers in Treg cells. Various T-cell subset data as the cell percentage in total lymphocytes and the marker expression levels of CD4+CD25 Treg cells between NPC patients and sex-and age-matched healthy control were statistically analysed using Mann–Whitney *U* test for nonparametric independent group comparisons.

### T-cell proliferation suppression assay

CD4+ T-cells were purified from PBMNC of five patients and four healthy blood donors with negative-immunomagnetic sorting using untouched CD4+ T-cell kit according to manufacturers' recommended protocol (Miltenyi, Germany). The cells were further positive-immunosorted using CD25 microbeads (Miltenyi) to isolate the CD4+CD25+ T-cell population. The CD4+CD25- T-cells were collected in the negative fraction. The CD4+CD25− T-cells (2 × 10^5^/ml) were plated onto BioCoat T-cell activation plates (BD Biosciences, Bedford, MA, USA). Autologous CD4+CD25+ T cells were added at ratios of 1 : 0, 1 : 1, 1 : 2, 1 : 5, and 0 : 1 to CD4+CD25− T cells. After 4 days, satisfactory cell viability was confirmed by using trypan blue exclusion method, and T-cell proliferation was assessed in triplicate by WST-1 cell proliferation assay (Roche, Germany).

## RESULTS AND DISCUSSIONS

### Reduced circulating CD4+ T cells in NPC patients

In this study, we first characterised the T-cell populations in PB of NPC patients. As shown in Table 1, the percentage of CD4+ (*P*=0.0321) but not CD8+ T cells were significantly decreased in NPC patients as compared with those in sex- and age-matched healthy controls. As the lymphocyte counts of NPC patients (1.7±0.6 × 10^9^/l) were similar to those in the controls (1.8±0.4 × 10^9^/l), this represents a reduction in CD4+ T-cell numbers in the PB of NPC patients. This was true for CD4+ cells in both memory CD4+CD45RO+ (*P*=0.0005) and naïve CD4+CR45RO− (*P*=0.0041) T-cell populations (Table 1).

### Expansion of circulating CD4+CD25^high^ regulatory T cells in NPC patients

**Figure 1:**
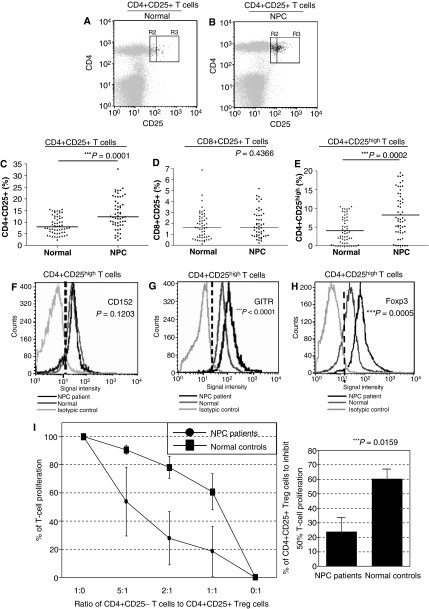
Characterisation of the Treg populations in PB of NPC patients and normal healthy blood donors. Gating of CD4+CD25+ (R2 and R3 gated regions) and CD4+CD25^high^ (R3 gated region) T cells of representative normal individual control (**A**) and NPC patient (**B**) are presented. The Foxp3-expressed T cells (coloured in black) were back-gated among the lymphocytes (coloured in grey) in the FASC plots. Percentages of circulating CD3+CD4+CD25+ T cells (**C**) and CD3+CD8+CD25+ T cells (**D**) in lymphocytes were determined in 56 NPC patients (▪) and 56 age- and sex-matched normal controls (▴).The CD3+CD4+CD25^high^ T-cell populations were also gated and the percentages between two groups were compared (**E**). The horizontal lines represent the mean values of the cell percentages. Expression levels of various Treg markers such as CTLA-4/CD152 (**F**), GITR (**G**), and Foxp3 (**H**) in the cells were also examined. Expression levels in terms of mean fluorescence intensity of these markers in representative cases of normal (yellow line) and NPC patients (green line) are shown. The differences in mean values of two groups were statistically analysed by Mann–Whitney *U* test and the significance level is set at 0.05. Asterisks indicate that the mean percentages of T-cell subsets in lymphocytes of NPC patients showed statistically significant differences as compared to those in controls. (**I**) Functional characterisation of circulating Treg in healthy volunteers (*N*=4) and NPC patients (*N*=5, four from the group of 56 patients and one was also analysed for TIL but not for PB Treg). CD4+CD25− T cells (2 10^5^/ml) were challenged with CD3 antibody and levels of cellular proliferation determined in the presence or absence of autologous Treg added at the ratios indicated. Mean levels of cellular proliferation are expressed as a percentage of that seen in cultures without the addition of Tregs. The mean percentages of CD4+CD25+ Treg, which can achieve 50% inhibition of autologous CD4+CD25− T-cell proliferation, were compared (right). The standard deviations of the percentages were presented as ‘T’ bars.

Despite this reduction in the number of circulating CD4+ T cells in NPC patients *vs* healthy donors, we found an increase in the subset of circulating CD4+ T cells that expressed CD25 (*P*=0.0001). No such increase was seen for CD8+CD25+ T cells (*P*=0.4366) (Table 1 and Figure 1A–D). A previous study has demonstrated elevated serum transforming growth factor-*β* (TGF-*β*) levels in NPC patients (Xu *et al*, 1999). Thus, it is of special interest to note that TGF-*β* can generate and expand CD4+CD25+ Treg from human PB (Yamagiwa *et al*, 2001). To exclude the population of activated T cells, which might express low levels of CD25 in human PB (Baecher-Allan *et al*, 2004), cells expressing high levels of CD25 were regated and quantified as Treg. Again, significant increase in CD4+CD25^high^ Treg was observed in the patients (Table 1 and Figure 1E). This increase did not correlate with NPC staging, tumour size, lymph node status, or the presence of metastasis (data not shown).

### Characterisation of circulating Treg in NPC patients

Using multiparametric flow cytometric analyses, we demonstrated that the majority (∼90%) of CD4+CD25^high^ cells in both NPC patients and controls expressed various intracellular and surface biomarkers characteristic of Treg. These biomarkers included the cytotoxic T-lymphocyte-associated antigen-4 (CTLA-4; CD152), the glucocorticoid-induced tumour necrosis factor receptor family-related protein (GITR), and the forkhead box transcriptional factor (Foxp3) (Table 1b). Expression of these markers by CD4+CD25^high^ cells strongly suggests that they are Treg. We also noted that levels of expression of GITR and Foxp3 were significantly higher in CD4+CD25^high^ cells from patients than controls (Figure 1F–H; Table 1b).

It has been demonstrated that Treg is anergic, and *in vitro* inhibition of CD4+CD25− T-cell proliferation by purified human CD4+CD25+ Treg is linked to their upregulation of *Foxp3* mRNA and protein (Baratelli *et al*, 2005). Thus, we further examined and compared the suppressive activity of CD4+CD25+ Treg cells in five NPC patients and four healthy volunteers. We confirmed the anergic nature of the Treg in both NPC patients and controls. More importantly, we found that the doses of Treg which could achieve 50% inhibition of autologous CD4+CD25− T-cell proliferation were significantly lower (*P*=0.0159) for NPC patients (23.89±9.44%) than for controls (60.40±6.87%) (Figure 1I). The increased suppressive activity seen in CD4+CD25+ T cells from NPC patients may reflect the greater number of CD4+CD25^high^ cells and/or the increased expression of Foxp3 and GITR in these cells compared with controls.

It was believed that CD4+CD25^high^ Treg belonged to the memory T-cell compartments (Dieckmann *et al*, 2001; Taams *et al*, 2001). However, we found that the expanded Treg not only exhibited CD45RO+ memory phenotype (∼70%), as in previous studies (17–18), but also CD45RO- naïve phenotype (∼30%) (Table 1a). Interestingly, Beyer *et al* (2006) recently also demonstrated an *in vivo* peripheral expansion of naïve CD4+CD25^high^ Foxp3+ Treg in multiple myeloma patients.

CD4+ T cells expressing the chemokine receptor CCR7 are able to migrate to secondary lymphoid organs and/or tissue (Bromley *et al*, 2005). Thus, to determine the migratory phenotype of the expanded circulating Treg in our NPC patients, we studied their expression of CCR7. Our data revealed that 93.7 and 92.2% of circulating Treg, respectively, in controls and NPC patients expressed similar levels of CCR7 (Table 1b), indicating a lymph node-homing capacity for the expanded Treg in our NPC patients.

**Figure 2:**
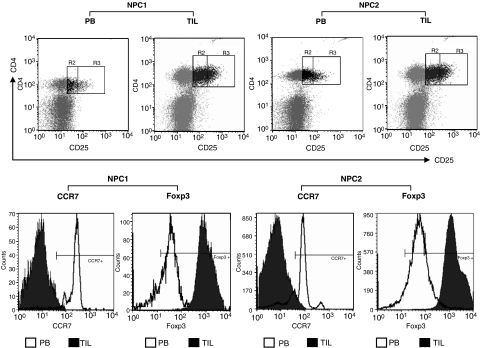
Characterising Treg in TIL and PB. The biopsy specimen was mechanically dissociated, fixed, and immunostained with the antibodies to CD4, CD25, and Foxp3 or CCR7. The suspended cells were analysed by flow cytometry gating on lymphocytes. Flow cytometric analysis on PB was as previously described in Figure 1. Two representative cases (NPC1 and NPC2) are shown. The percentage of Tregs was explored in TIL(▪) and PB (□) analysing the CD4+CD25+ gated population (R2 and R3) and the CD4+CD25^high^ population (R3 alone). Expression of Foxp3 and CCR7 in CD4+CD25^high^ Treg from both TIL and PB are presented as histograms. For these histograms, they are the composite of two different FACS plots, one from the data of PB (white coloured peak) and another from TIL (black coloured peak), highlighting the loss of CCR7 expression and increased Foxp3 expression in the CD4^+^CD25^high^ of TIL as the shifts of those peaks. The horizontal line represents the cutoff of positivity defined using an isotype-matched control antibody.

To determine if Treg infiltrated the tumour site, we examined the tumour infiltrating lymphocytes (TIL) in NPC biopsies from five patients. We found that 10.96±1.09% of TIL exhibited a CD4+CD25^high^Foxp3+ Treg immunophenotype. Interestingly, levels of Foxp3 expression in CD4+CD25^high^ TIL were considerably higher than those seen in circulating CD4+CD25^high^ cells. Furthermore, CD4+CD25^high^ TIL did not express CCR7. (Figure 2; Table 2). The relatively low numbers of TIL that can be obtained from NPC biopsies did not allow functional tests on this population, but it is interesting to speculate that elevated levels of Foxp3 expression correlates with increased suppressive activity. The absence of CCR7 indicates that the migratory properties of these cells has been changed, so that they no longer home to lymph nodes but now migrate to the tumour tissue

In conclusion, we have demonstrated an increase of Foxp3+CD4+CD25^high^ Treg in PB and tumour sites in NPC patients. The expanded Treg in the circulation also showed enhanced suppressive activity on CD4+CD25− T-cell proliferations. The increase of this functional Treg population might reduce T-cell-mediated antitumour immunity as represented by the significant decrease in CD4+ T-cell populations in the NPC patients. Thus, the present findings have provided important information and insight into the future design of immunotherapeutic strategies for NPC.

## Figures and Tables

**Table 1:** Characterization of T-cell populations in the peripheral blood of 56 NPC patients and 56 age- and sex-matched healthy blood donor controls

**(*N*=56)**	**Normal (%)^a^**	**NPC (%)^a^**	***P*-value**		
*(a) T-cell subsets*					
CD3+CD4+^b^	***21.7***±***9.9***	***17.8*** ±***10.1***	***0.0321***		
CD3+CD8+^b^	13.2±6.7	12.0±7.0	*0.3130*		
CD4+CD45RO+	**18.2±8.5**	**12.6±6.7**	***0.0005***		
CD4+CD45RO-	**9.4±5.7**	**6.9±6.0**	***0.0041***		
CD4+CD25+	**8.8±3.8**	**13.6±6.7**	***0.0001***		
CD4+CD25^high^	**4.0±3.6**	**8.2±5.9**	***0.0002***		
CD4+CD25^high^CD45RO+	**2.7±2.4**	**6.4±5.1**	***<0.0001***		
CD4+CD25^high^CD45RO−	**1.3±1.6**	**2.6±2.7**	***0.0052***		

	**Cells with positive expression**		**Signal levels**		
**(*N*=56)**	**Normal (%)**	**NPC (%)**	**Normal (MFI)**	**NPC (MFI)**	***P*-value**
*(b) Treg marker expressions in circulating CD4+CD25*^*high*^ *cells*					
CD4+CD25^high^ CTLA4/CD152	87.6±11.2	94.6±13.3	406±31	420±28	*0.1203*
CD4+CD25^high^ GITR	99.9±0.3	98.0±13.5	**526±68**	**606±48**	***<0.0001***
CD4+CD25^high^ Foxp3	90.9±8.1	94.2±13.7	**522±147**	**602±110**	***0.0005***
CD4+CD25^high^ CCR7	93.7±7.2	92.2±20.6	414±42	423±48	*0.2443*

a Denotes % in total lymphocytes.

b The CD4/CD8 ratio in NPC patients (1.2±0.4) was significantly reduced as compared with that of normal controls (1.9±0.9) (*P*<0.0001).

Bold prints in % and *P*-values represent statistically significant differences.

MFI refers to mean fluorescence intensity.

NPC=nasopharyngeal carcinoma.

**Table 2:** Characterisation of tumour infiltrating lymphocytes (TIL) in five NPC patients

**TIL (*N*=5)^a^**	**NPC (%)**
CD4+CD25+	32.24±7.59%^b^
CD4+CD25+Foxp3+	30.96±7.18^b^
CD4+CD25+Foxp3+CCR7−	86.42±8.79^c^
CD4+CD25^high^	12.37±2.45%^b^
CD4+CD25^high^Foxp3+	10.96±1.09^b^
CD4+CD25^high^Foxp3+CCR7−	84.98±11.79^d^

a Four patients were from the group of 56 with PB Treg data and one was also analysed for T-cell proliferation suppression assay, but with no PB Treg data.

b % in TIL.

c % in CD4+CD25+ Treg.

d % in CD4+CD25^high^ Treg.
